# Negative pressure therapy in pediatric patient with surgical site infection: experience report

**DOI:** 10.1590/1980-220X-REEUSP-2024-0077en

**Published:** 2024-11-11

**Authors:** Leticia Pontes, Juliana Szreider de Azevedo, Izabela Linha Secco, Higor Pacheco Pereira, Solange Cristina Moreira Vieira, Regiane Queiroz Afonso

**Affiliations:** 1 Universidade Federal do Paraná, Programa de Pós-Graduação em Prática do Cuidado em Saúde, Curitiba, PR, Brazil.; 2 Hospital Infantil Doutor Waldemar Monastier, Campo Largo, PR, Brazil.

**Keywords:** Surgical Wound Infection, Negative-Pressure Wound Therapy, Wound Healing, Nursing Care, Pediatrics, Infección de la Herida Quirúrgica, Terapia de Presión Negativa para Heridas, Cicatrización de Heridas, Atención de Enfermería, Pediatría

## Abstract

**Objective:**

To describe the use of negative pressure wound therapy and hydrofiber dressing with silver in a pediatric patient with a hard-to-heal surgical wound infection.

**Method:**

This is a descriptive professional experience report on the use of conventional dressings and negative pressure wound therapy in a pediatric patient with a surgical wound infection. It was developed in 2023 at a Public Health Service that is a reference in the care of pediatric patients in the state of Paraná.

**Results:**

The surgical wound dehiscence started 12 days after peritoneostomy. Initially, the wound was treated with hydrofiber dressing with silver for 22 days and subsequently, negative pressure wound therapy was used for 15 days, regenerating the wound.

**Conclusion:**

Negative pressure wound therapy in pediatrics proved to be safe, effective and efficient for the treatment of complex wounds and corroborated the skin regeneration process, as did hydrofiber dressing with silver.

## RESUMEN

**Objetivo:**

Describir el uso de la terapia de presión negativa para heridas y el apósito de hidrofibra con plata en un paciente pediátrico con infección de herida quirúrgica de difícil cicatrización.

**Método:**

Se trata de un informe descriptivo de experiencia profesional sobre el uso de apósitos convencionales y terapia de presión negativa para heridas en un paciente pediátrico con infección de herida quirúrgica. Se realizó en 2023 en un Servicio Público de Salud que es referencia en la atención a pacientes pediátricos en el estado de Paraná.

**Resultados:**

La dehiscencia de la herida quirúrgica se inició a los 12 días de la peritoneostomía. Inicialmente, se trató la herida con apósito de hidrofibra con plata durante 22 días y posteriormente se utilizó la terapia de presión negativa durante 15 días, regenerando la herida.

**Conclusión:**

La terapia de presión negativa para heridas en pediatría demostró ser segura, eficaz y eficiente para el tratamiento de heridas complejas y corroboró el proceso de regeneración de la piel, al igual que el apósito de hidrofibra con plata.

## INTRODUCTION

Toxic megacolon (TM), an intestinal complication that causes colon distension, is fatal if not diagnosed early, as it presents systemic manifestations of toxicity^(1)^. It is mainly triggered by inflammatory bowel diseases (IBD) such as ulcerative colitis (UC) or Crohn’s disease (CD)^(2)^, but also by other less frequent etiologies such as chronic intestinal constipation, *Clostridium difficile* infection, among others^(3,4)^. Although the first-line clinical treatment is medication, surgical treatment is needed in emergency situations such as intestinal perforation, necrosis, full-thickness ischemia of the wall and peritonitis^(4)^.

Surgical site infection (SSI) ranks third among Healthcare-Associated Infections (HAIs), affects 14-16% of hospitalized patients and can be evidenced up to 30 days postoperatively^(5,6)^. As gastrointestinal surgeries explore cavities that are already colonized, they are considered potentially contaminated and have a higher risk of SSI^(7)^. If the surgical wound is contaminated and presents suture dehiscence, it is characterized as a hard-to-heal wound^(8)^, and negative pressure wound therapy (NPWT) is indicated to aid in its treatment^(9)^.

Negative pressure wound therapy began to be used in the late 1980s to treat chronic and infected wounds. It generates negative pressure by means of a hydrophobic polyurethane foam and a sterile transparent plastic film applied to the wound bed and edges, connected by a plastic tube to a vacuum. This type of dressing reduces edema and drains excess exudate, promotes debridement, accelerates perfusion, vascularization and the formation of granulation tissue^(10,11)^.

Since there are few multicenter randomized studies on the use of NPWT in pediatrics, more research on the subject is needed to prove its effectiveness in comparison to other existing therapies, and thus support nurses’ decision-making when choosing the best therapy for treating SSI injuries. Because of its high cost and lack of evidence, this technology is still not used in tertiary health institutions of the Health Department in the state of Paraná.

Therefore, the objective of this experience report is to describe the use of negative pressure wound treatment and hydrofiber dressing with silver in a pediatric patient with a hard-to-heal surgical wound infection.

## METHOD

### Study Design

The Standards for Reporting Qualitative Research (SRQR) was the tool adopted in the development of this report^(12)^.Experience reports do not come from studies. Their purpose is to describe the experience of an individual or a group of professionals in a given situation. The use of the SRQR is suggested as a guidance for writing the experience report article, since there is still no specific guideline for this purpose^(13)^.

### Location

The experience reported occurred in a children’s hospital in the metropolitan region of Curitiba, Paraná. This is a public state institution that assists patients between the ages of zero days and 17 years from all over the statevia the Bed Regulation Center. The hospital in question treats cases of medium and high complexity and has 112 active beds distributed among neonatal intensive care units (ICU) (20 beds) and pediatric intensive care units (20 beds), 47 clinical ward beds and 25 surgical ward beds. It also includes a surgical center with three operating rooms and a medical outpatient clinic for pediatric specialties (low- and high-risk childcare, cardiology, pulmonology, nephrology, orthopedics, dermatology, ophthalmology, gastroenterology, nutrology, otorhinolaryngology, neurology, neurosurgery, and vascular, plastic, and pediatric surgery).

The Pediatric ICU, the setting for this report, has a multidisciplinary team composed of nurses, nursing technicians, physicians, resident physicians, physiotherapists, a nutritionist, a social worker, an occupational therapist, a speech therapist, and a psychologist to provide comprehensive patient care.

In 2023, the Pediatric ICU had 545 admissions (25 discharges, 491 internal transfers, 19 external transfers, and 10 deaths). The occupancy rate was 83%, the average length of stay was 11.7 days, and the infection rate was 19%, with 11 central line-associated bloodstream infections (CLABSIs), six ventilator-associated pneumonias (VAP), three urinary tract infections (UTI) with indwelling bladder catheters, and nine others; and the mortality rate was 3.17%. Respiratory failure was the most prevalent diagnosis.

### Population and Selection Criteria

The patient was selected for this report because he had a diagnosis of TM, underwent a gastrointestinal surgical procedure, developed SSI, and presented with hard-to-heal surgical wound dehiscence, which culminated in the indication for NPWT.

### Data Collection

The data collection period was from June 2023 to July 27, 2023. The electronic medical record was used to search for information on the patient’s clinical progress, diagnoses, and nursing care. The nurses on the Institution’s Skin Care Committee prepared a Word document to record the progress of the healing process. This report was timely, as the care provided was described in a single document, providing faster access for assessment and new measures to be taken. It contained a visual description of the wound, its measurement, photographic record, and the dressing used to treat the wound. These data and images were chronologicallytranscribed into the document by the nurses, while the Bates-Jensen Wound Assessment Tool (BWAT) scale and chart were transcribed into Excel. The mother, who remained by the patient’s side throughout the hospitalization, was active in the process of caring for the wound, as was the patient after being extubated.

### Data Analysis and Treatment

The clinical progress and nursing diagnoses were analyzed from the electronic medical record. The progress of the healing process and the choice of treatment were analyzed by reading the description, image, and graphs contained in the Word and Excel reports. The predictors of the tool (BWAT) were used to score the characteristics of the wound, and using data from the graph, nurses could visualize if the wound was progressing in the healing process or not. The photographic record also allowed the visual analysis of the characteristics and reduction of the wound dimensions. The analysis was performed objectively, describing the wound and the use of conventional dressing and NPWT.

### Ethical aspects

The project was approved by the Research Ethics Committee of the Complexo Hospital de Clínicas – Universidade Federal do Paraná, according to substantiated opinion number 6.159.293 of July 3, 2023, respecting the ethical precepts of Resolution No. 466/2012 of the National Health Council. The patient’s legal guardian authorized the capture and dissemination of the photos after signing the Image Consent Form.

## RESULTS

### Experience Report

Patient P.H.F.G., seven years old, diagnosed with TM after colon biopsy in 2021. During his hospital stay before admission to the study site, he underwent 15 exploratory laparotomiesbetween 2021 and 2023.

He was admitted to the pediatric ICU of the children’s hospital on June 1, 2023 with severe hydroelectrolytic disorder due to losses through the stoma. He was wearing a Bogota bag, fasting, without Total Parenteral Nutrition (TPN), under high invasive mechanical ventilation parameters, open nasogastric catheter with large amounts of bilious output. Presence of double-lumen central venous access in the right subclavian vein, continuous sedation and analgesia with fentanyl, midazolam and precedex.

Abdomen with open surgical wound, peritoneostomy and Bogotabag, cavity showing intestinal content and serosanguineous exudate; edematous colostomy on the left side and Penrose drain in the lower right flank with serous output, significant scrotal edema and diuresis control with indwelling urinary catheter. Cold extremities, capillary refill time of two seconds, hemodynamically stable. Braden Q scale equivalent to moderate risk (13), Fugulin classification scale for intensive care (44). The patient was promptly evaluated by the pediatric surgery team, who decided on a new surgical approach to clean the abdominal cavity, create a new colostomy, close the peritoneostomy and insert a new central vascular access device.

To cover the retroabdominal surgical wound, which started at the xiphoid process and ended at the pubic symphysis, exudate absorption foam was chosen with an expected change at every two days if saturated. On June 7, 2023, the patient had a cardiorespiratory arrest lasting three minutes, which was reversed. The patient began using vasoactive drugs (VAD).

**Figure 1:**
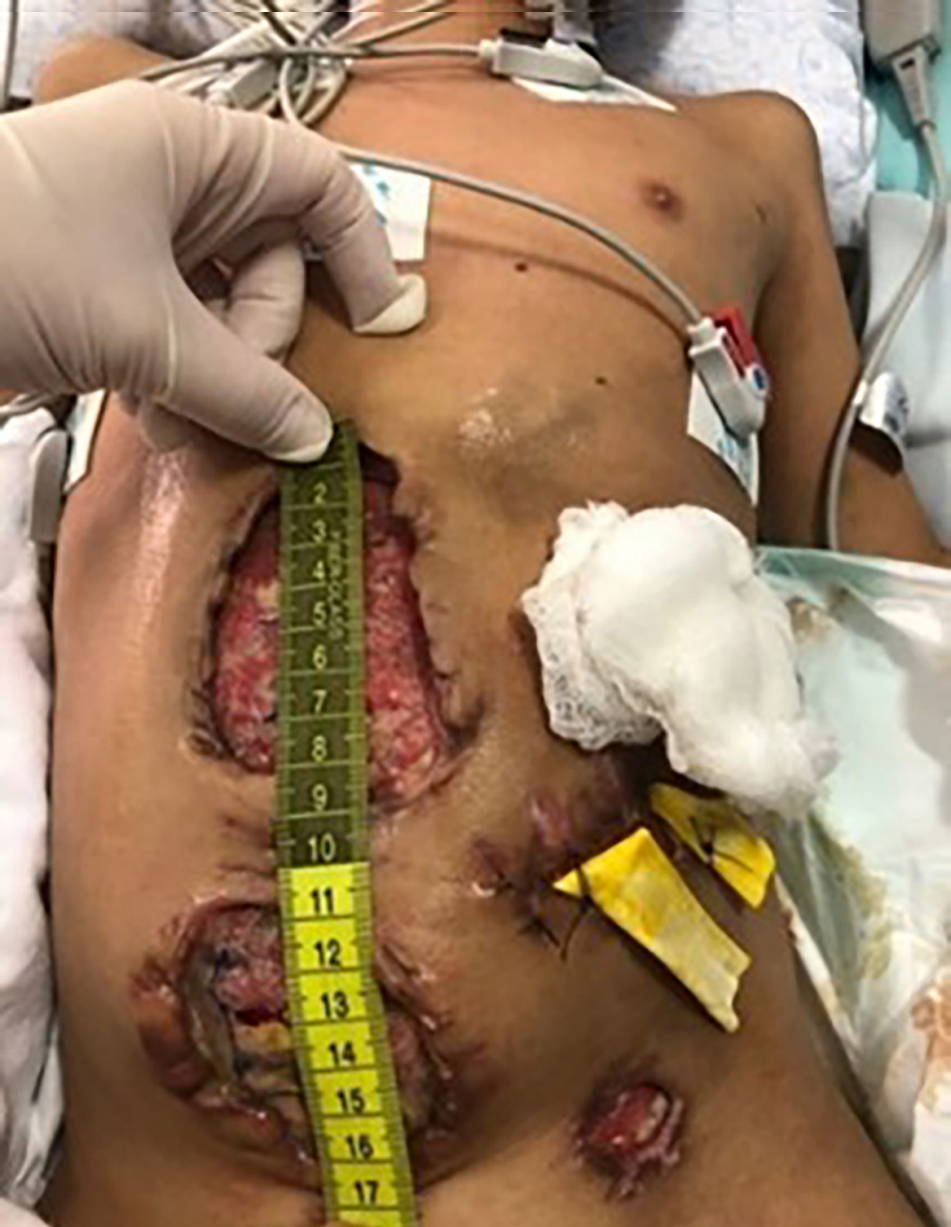
Immediate postoperative period of abdominal revision on July 19, 2023.

After 12 days, with signs of SSI, the patient presented suture dehiscence that reached the entire upper and lower part of the umbilical scar. A surgical approach was performed with revision of the abdominal cavity, cleaning of clots and debridement of the surgical woundedges, but there was no approximation of the edges, which were detached, tending to epiboly. Muscle, adipose and granulation tissue were visible in the wound bed. The skin was intact around the wound. The abdomen was still edematous and tense. The surgical wound in the lower left quadrant below the colostomy was sutured and had two Penrose drains. The therapeutic conduct and nursing care were as follows: cleaning and decontamination using gauze soaked in polyhexamide cleaning solution, Prontosan^®^. The wounds were then filled with hydrofiber dressing with silver and exudate absorption foam. This dressing was changed every two days, ending on July 12, 2023. The dimensions of the wounds on June 19, 2023 were: upper surgical wound 10 cm × 7 cm; lower 6 cm × 6 cm (Figure 1). On that day, the patient was still hemodynamically unstable, using VAD with infusion of TPN, under invasive mechanical ventilation in pressure-volume controlled mode with controlled pressure of 21, inspired oxygen frequency of 90%, and oxygen saturation of 90%.

**Figure 2:**
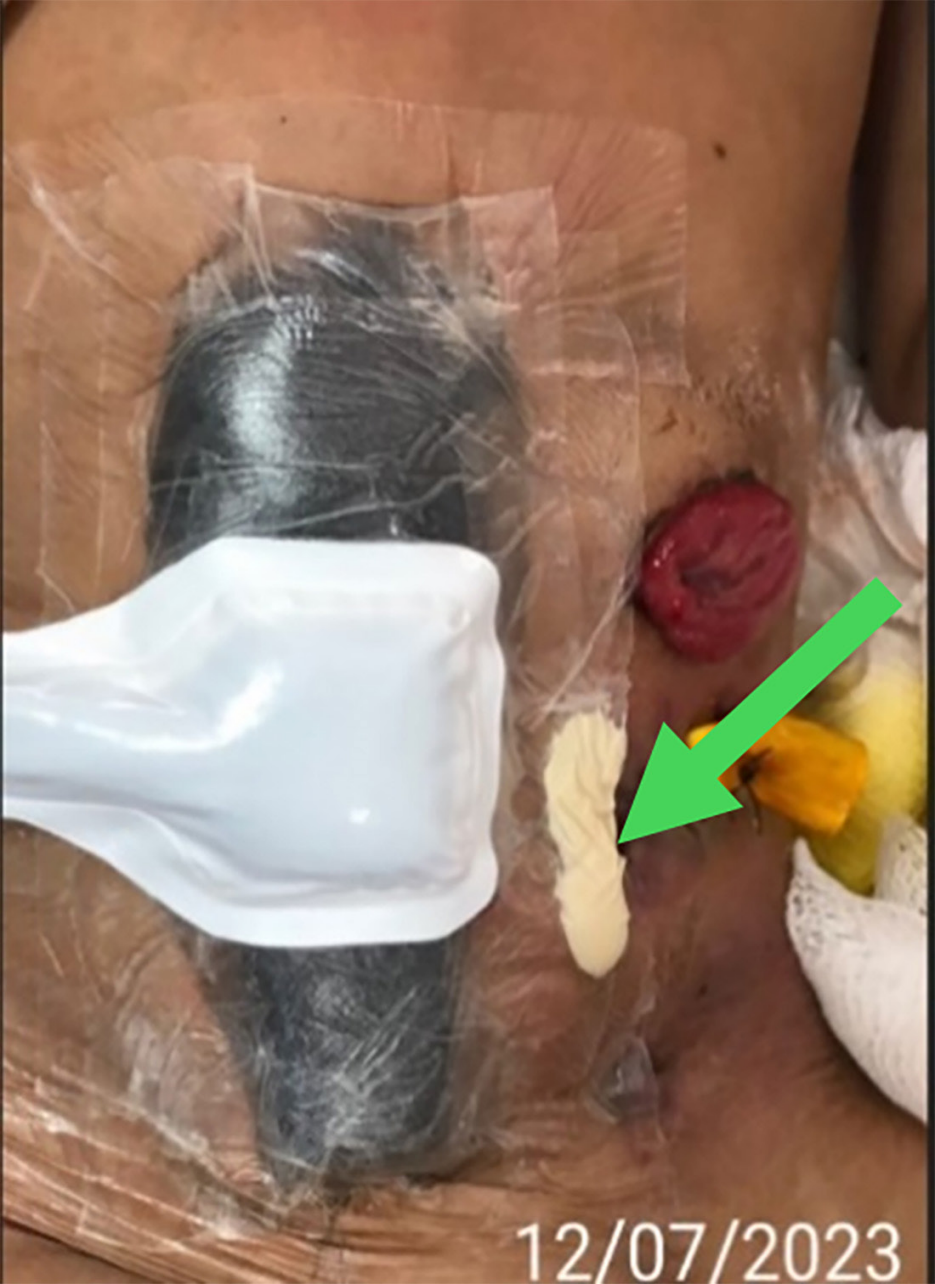
Clean wound bed and application of negative pressure therapy. The arrow indicates the hydrocolloid paste.

On December 12, 2023, after discussion, guidance and explanation to the patient, his guardian and the multidisciplinary teamabout the NPWT innovative technology, the decision to use it was made. The patient was now extubated, fasting,under oxygen therapy via nasal catheter and infusion of TPN. The materials were donated for one month of treatment with changes estimated at every four days. The sequence of NPWT application occurred according to the following steps: a) cleaning the wound bed with gauze soaked in Prontosan^®^ acting for ten minutes; b) placement of non-adherent gauze (Jelonet) to protect the injured tissue and prevent contact of the tissue with the foam; c) application of the foam in order to join the upper and lower wounds; d) occlusion with Opsite^®^ transparent and impermeable film; e) application of the Soft Port; f) connection to the compressor at a pressure of −100 mmHg. The surgical wound with the Penrose drain caused a bulge in the skin, which needed to be sealed with hydrocolloid paste (Figure 2, green arrow). In this surgical wound, there was still extravasation of a large amount of fecal fluid due to the fistula, with the skin around and below showing erosive injury of the epidermis. Skin protection powder and barrier spray were used throughout this skin area. The patient did not report pain or any discomfort during application of this technology, nor with the vacuum. For the nursing team, it was a challenge to maintain the integrity of the Opsite^®^ adhesion to the skin given the liquid content of the fistula. Wound dimensions on July 12, 2023: upper wound 6 cm × 3 cm; lower wound 4 cm × 3 cm.

**Figure 3:**
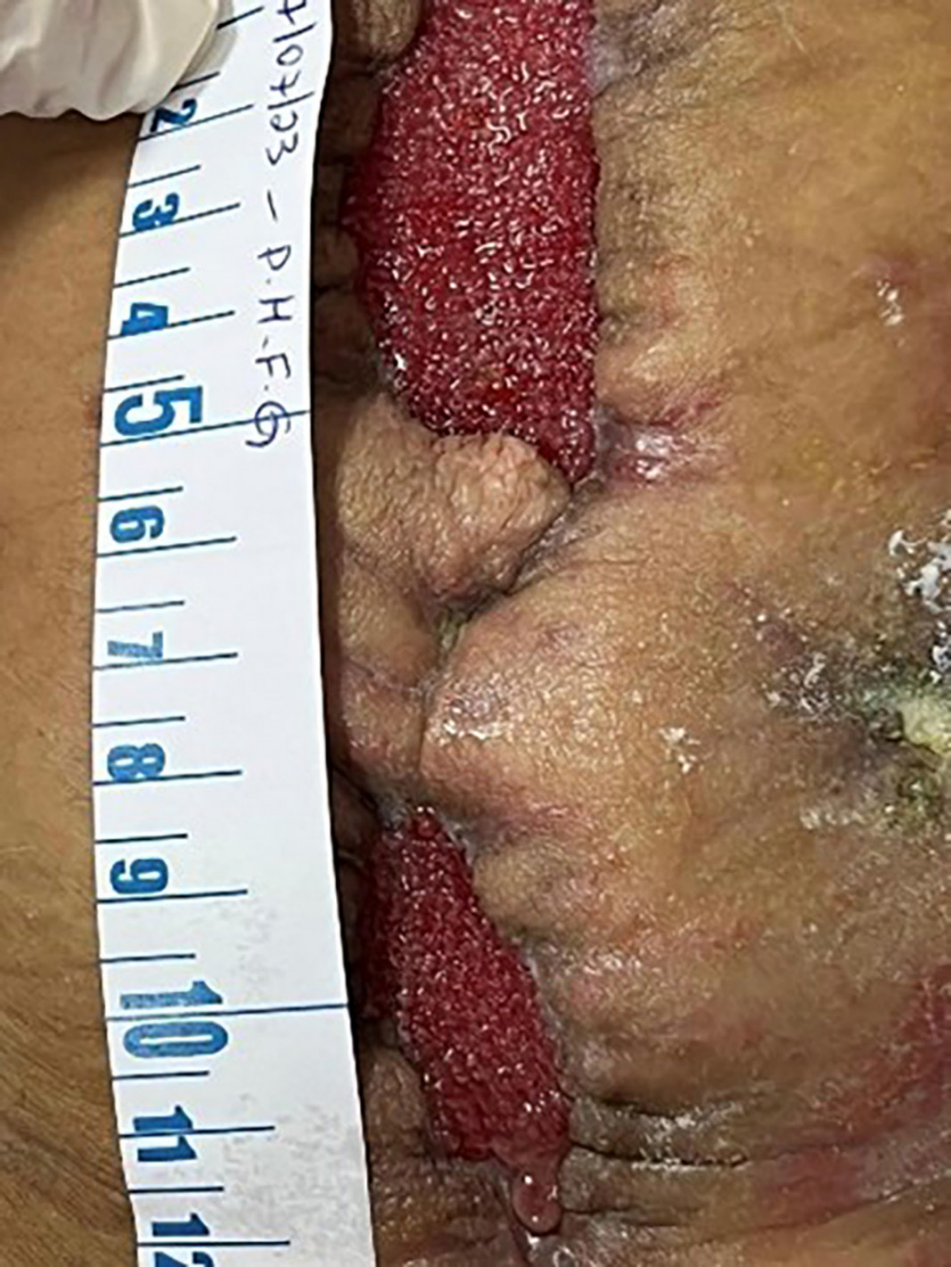
Wound after cleaning with Prontosan^®^ with granulation tissue and adhered edges.

The second change of the NPWT system was performed on July 17, 2023, and the wound had the following dimensions: upper wound 5.5 cm × 2.8 cm; lower wound 4 cm × 2 cm. The entire lower left abdominal quadrant had an increase in the eroded area due to dermatitis associated with the enteric effluent from the fistula. A light bath was started for half an hour a day after the bath with subsequent application of skin protection powder with a barrier spray. There were granulation tissue and adhered edges in the wound bed (Figure 3). It was necessary to apply hydrocolloid paste to the entire left lateral edge of the Opsite^®^ to block the infiltration of feces from the fistula.

The third change was performed on July 21, 2023: upper wound 5 cm × 2 cm and lower wound 4 cm × 1 cm. The upper wound bed remained with 100% granulation tissue, the lower wound bed was pale, as there was infiltration of feces under the Opsite^®^ and contamination of the wound.

**Figure 4:**
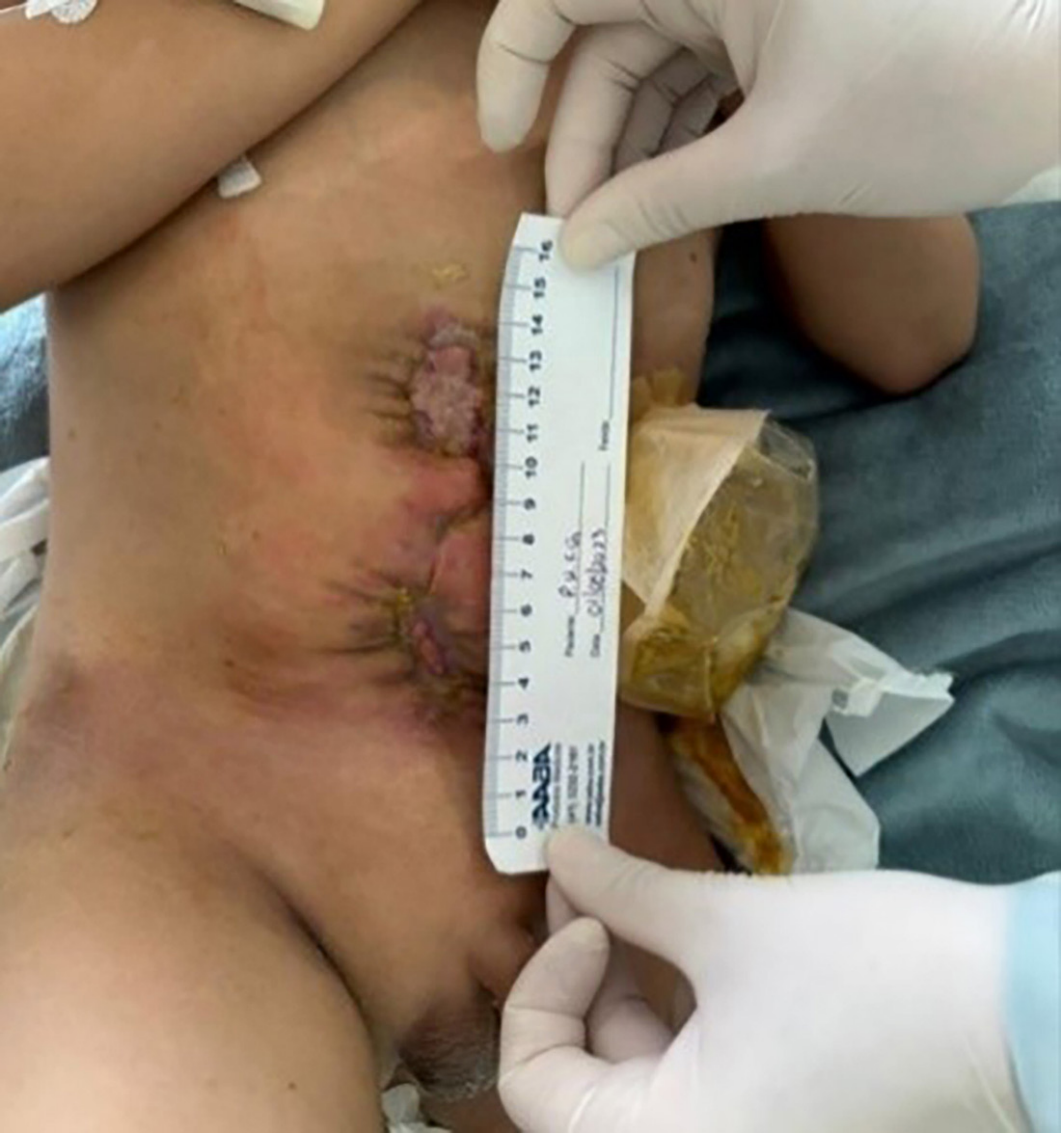
End of negative pressure therapy.

The last change of the NPWT system was on July 25, 2023. The dressing was removed with an extensive dirty area contaminated by fecal effluent. The surrounding skin showed an increased area of hyperemia and erosion of the epidermis, making the adhesion of the transparent film difficult. Upper wound: 4 cm × 1.8 cm, with pale bed, regular and adhered edges. Lower wound: 3 cm × 1 cm, pale bed with islands of hypergranulation, regular and adhered edges to the bed. Two days later, the transparent film completely detached from the skin due to excess moisture and discontinuity of the epidermis, bringing forward the end of the therapy. The final dimensions were: 2.5 cm × 1 cm in the upper wound and 2 cm × 0.5 cm in the lower wound. Both presented an opaque bed and hypergranulation, adhered and regular edges (Figure 4). The total volume of exudate aspirated using the technology throughout the treatment was 60 mL.

Continued wound care included cleaning with Prontosan^®^, primary dressing with silver exudate transfer foam, and secondary dressing with gauze and micropore. The surrounding skin remained covered with skin protection powder and barrier spray. The patient progressed with a Braden Q scale of 17, compatible with mild risk, and a Fugulin scale compatible with moderate care, with a nasogastric tube diet and intravenous TPN infusion.

The patient was discharged from the pediatric ICU to the clinical ward on August 16, 2023 and discharged from hospital two days later, totaling 79 days of hospitalization.

**Figure 5:**
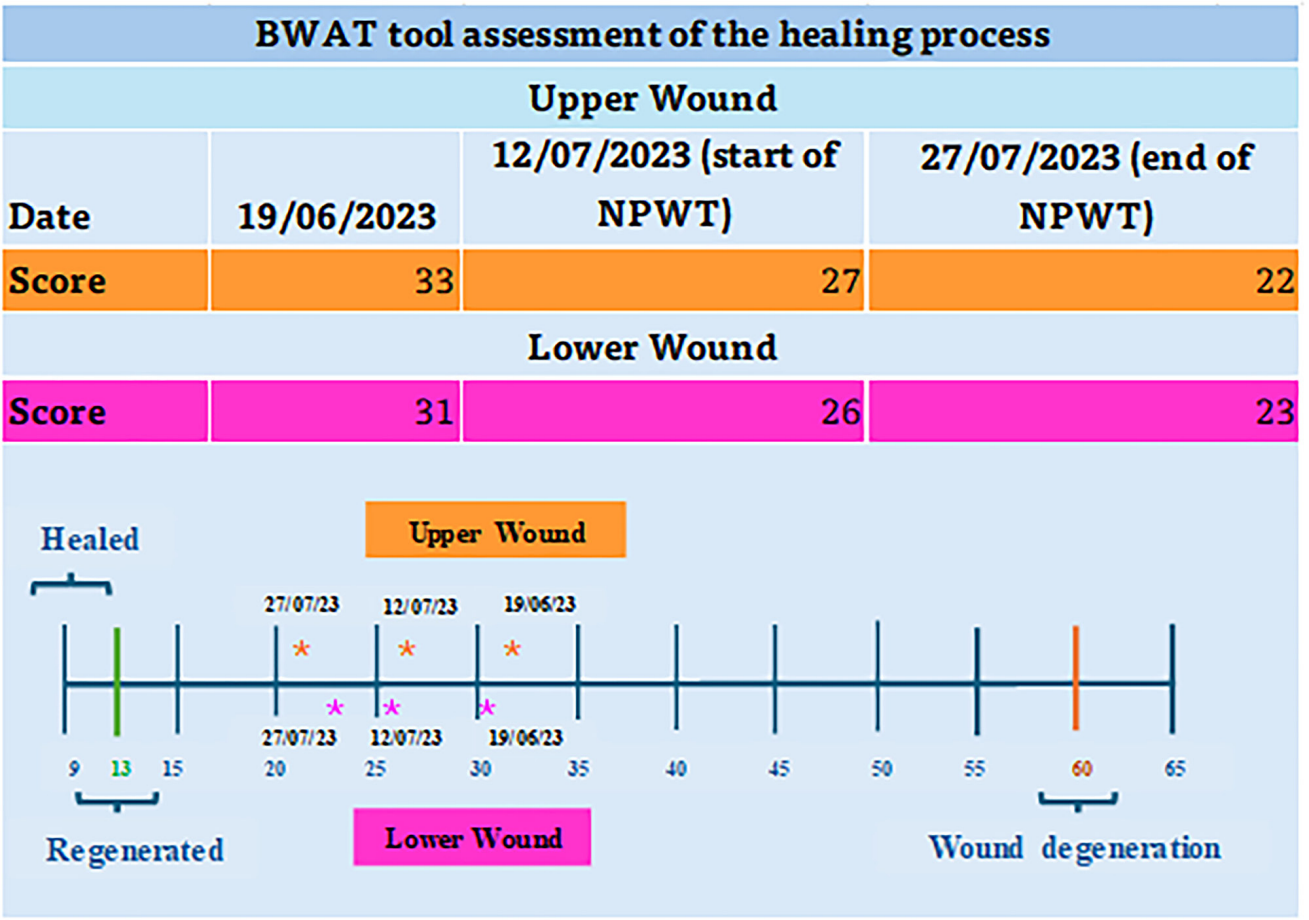
Bates-Jensen tool for wound management.

The healing process was managed using the BWAT tool, as shown in Figure 5. This figure contains the total score, corresponding to the sum of the score for each predictor of assessment of 13 wound characteristics.

## DISCUSSION

Measures for the prevention and control of SSIs are widely discussed and developed by competent bodies at the national level and in institutions. Particularly in institutions, these measures are still not very evident in professional practice, and decision-making on the appropriate management of these complications is mandatory in the nursing care process, since these are hard-to heal wounds^(14)^.

Some actions should be valued to provide complication- free nursing care in potentially contaminated surgeries. Nursing interventions that contribute to reducing SSIs in this surgical profile can be classified into three stages: in the preoperative period, with the use of prophylactic antibiotic therapy, chlorhexidine alcoholic bath and hand hygiene; in the intraoperative period, with sterile attire, change of sterile gloves, use of a pack to close the fascia and skin, disinfection and antisepsis, surgical classification as potentially contaminated, surgical time, antibiotic redosing, body warming and capillary blood glucose; and in the postoperative period, again bathing with chlorhexidine disinfectant and hand hygiene, care with dressings and drains, temperature and blood glucose control, use of sterile gauze in individual packages and patient education with guidance during hospitalization and discharge^(15)^. In this report, the multiple surgical approaches to the patient, the open surgical wound and the release of fecal matter contaminating the wound corroborated the high probability of SSI. After assessing the risk, it is important that nurses choose the best dressing and apply it to provide a favorable environment for healing.

Since Florence Nightingale, wound care has become professionalized and accumulated more than 150 years of scientific knowledge related to healing. The complexity of wounds has increased in the same proportion as the technological advances in dressings and, therefore, the vast possibilities of dressings can raise doubts in nurses. The basic premise is to know that in order to create an optimal healing environment, these professionals must understand the healing phase, the characteristics of the wound and the purpose of each dressing. For better assertiveness in wound care, the decision-making process (evaluation, diagnosis, intervention and management of the wound with standardized scales) should be restricted to a professional who is properly trained and qualified for this purpose^(16,17)^. However, even for qualified professionals, the search for evidence on the subject is limited in pediatric patients, culminating in doubts about the most effective therapy^(17)^.

The physiological process of healing occurs in four phases: hemostasis, inflammation, proliferation and remodeling. Although all stages are important in the healing event, in the inflammatory phase there is an agglomeration of macrophages and neutrophils that generate exudate and protect the wound from undesirable microorganisms and maintain the environment favorable for the proliferation of new cells, skin remodeling and healing^(17)^. The choice of dressing depends on the healing phase and will change according to its evolution. The purpose of the dressing is to keep the environment moist, warm, clean, and free from bacterial invasion. It should be easy to remove (atraumatic) and not require frequent changes in order to provide stability to the tissues and faster remodeling^(18,19)^. Since the patient was extubated only at the beginning of the use of NPWT, it was from this moment that he gave his report on atraumatic and painless dressing changes.

One of the most common reasons for impeding the natural healing process is infection and the presence of biofilm. Its formation is complex, involves different phases and depends on the type of microorganism involved. The bacteria present in the wound bed begin to proliferate, adhere and excrete extracellular polymeric substance, which forms the biofilm and facilitates the proliferation of these bacteria, accelerating their maturation phase and making them ready to colonize other areas^(20)^. This complex system allows not only physical protection, but also gene transfer, through which the sensitivity to select distinct properties that promote greater protection, aid intercellular communication and encourage the growth of beneficial species is transmitted to the gene. In other words, characteristics that facilitate the genetic improvement of bacteria, making them more resistant to the environment and antibiotics, resulting in hard-to heal wounds^(21)^.

An in-vivo study compared the use of hydrofiber – alone; hydrofiber with silver – alone; hydrofiber + cleaning solution composed of Octenisept^®^; and hydrofiber + Prontosan^®^ and their action in controlling biofilm. It was proven that the cleaning solutions eliminate and/or significantly reduce the microbial load. The same result was also observed when compared to the use of hydrofiber with silver^(22)^. Prontosan^®^, composed of purified water, polyaminopropyl biguanide (polyhexamide, PHMB) 0.1%, which is a synthetic antimicrobial peptide, acts by breaking down the glycosaccharide wall of the bacterial cell wall, destroying the bacteria. Betaine 0.1%, contained in the product is a hydrophilic and hydrophobic surfactant with the ability to perform wound debridement^(23)^.

In addition to cleaning and antisepsis of the wound bed in infected surgical wounds, hydrofiber dressings with silver promote ideal hydration of the environment, absorbing excess exudate, while the silver acts as an antimicrobial. This dressing also has the benefit of not needing frequent changes^(21)^. The use of these products helps to reduce the local microbial load, maintaining the environment conducive to healing, even in injuries originating from potentially contaminated surgical wounds.

Although some therapeutic modalities are specialized to treat SSI, they sometimes need to be replaced by superior technologies, since the presence of biofilm slows down the healing process. Therefore, NPWT has been an innovative and adjuvant therapeutic route in the treatment of hard-to-heal wounds. Although the World Health Organization recommends the prophylactic use of NPWT for the prevention of SSI in the immediate postoperative period of primary-intention surgical wounds^(24)^, its use in clinical practice is commonly observed in postoperative complications that progressed with SSI^(24)^.

The mechanism of action of NPWT with pressures of up to −200 mmHg intermittently or continuously in the wound bed reduces excess exudate, edema, inflammation and cellular debris. This action reduces the bacterial load and improves blood and lymphatic flow, allowing neovascularization, improving the supply of oxygen and nutrients to the cells, forming granulation tissue and favoring epithelialization and consequent healing^(25,26)^.

The significant outcomes of the use of NPWT are the preexisting adverse conditions of the wound before its adoption, such as SSI, bleeding, cellulitis/abscess, septicemia and fistula, which totaled 63.4%. After installation, these complications reduced drastically to 6.2%^(27)^. The financial burden to the institution (professionals, facilities, insurance reimbursement, patient out-of-pocket expenses) before and after NPWT totaled, respectively, $11,029.01 and $3,202.43 per month^(9,24)^. In the present report, a fistula appeared prior to the initiation of therapy, which confirms the evidence from the reported studies and how the presence of the fistula was a challenging factor in maintaining a proper adhesion of the vacuum dressing to the patient’s skin.

Therefore, the conclusion is that the NPWT is an innovative and safe technology for use in pediatrics and contributes to optimizing the healing of infected surgical wounds.

### Study Limitations

The evidence presented in studies on the treatment of SSI using NPWT in pediatric abdominal surgeries is scarce. The vast majority of studies were conducted in adults. This study was based on the analysis of a single scenario and a single sample, since the equipment was donated for exclusive use by this patient for one month. Therefore, this experience report is important to encourage nurses to conduct case-control studies on the use of NPWT versus conventional therapies in other pediatric institutions.

### Advances for Nursing

High-level scientific production on NPWT performed by pediatric nurses is increasingly necessary and will support other professionals in their decision-making. This evidence, once its effectiveness has been proven, can help managers motivate the standardization and purchase of this equipment that assists in the healing process. This experience report reveals nurses’ protagonist role and autonomy in skin and wound care, making them executors of Advanced Nursing Practice as through the search and generation of evidence, they are in a differentiated condition to make decisions, adopt assertive conducts and assist patients who depend on them for recovery.

## CONCLUSION

The initial treatment with hydrofiber dressing with silver lasted 22 days, with a six-point reduction on the BWAT scale in the upper wound and five in the lower wound (BWAT 33 and 31 to 27 and 26, respectively). The NPWT was the auxiliary therapy used for 15 days, with a reduction of five points on the BWAT scale in the upper wound and three points in the lower wound (BWAT 27 and 26 to 22 and 23, respectively). The NPWT proved to be safe, effective and efficient, providing comfort and reducing stress to the patient, due to more spaced changes, thus improving the patient’s experience in the care for complex wounds.

The BWAT tool assists in managing the healing process. Each predictor of the wound characteristics varies between one-five points that added together vary from nine (wound regeneration) to 65 (degenerated wound). In this report, all scores indicated the proximity of regeneration.
