# Supplementary material for: Negative pressure therapy in pediatric patient with surgical site infection: experience report
Source: Rev Esc Enferm USP. 2024 Nov 11;58:e20240077. doi: 10.1590/1980-220X-REEUSP-2024-0077en (PMC11584165; doi:10.1590/1980-220X-REEUSP-2024-0077en)
Supplement: Translation

# Translation: Terapia por pressão negativa em paciente pediátrico com infecção de sítio cirúrgico: relato de experiência

**DOI:** 10.1590/1980-220X-REEUSP-2024-0077pt

**Authors:** Leticia Pontes, Juliana Szreider de Azevedo, Izabela Linha Secco, Higor Pacheco Pereira, Solange Cristina Moreira Vieira, Regiane Queiroz Afonso

**Affiliations:** 1 Universidade Federal do Paraná, Programa de Pós-Graduação em Prática do Cuidado em Saúde, Curitiba, PR, Brasil.; 2 Hospital Infantil Doutor Waldemar Monastier, Campo Largo, PR, Brasil.

**Keywords:** Infecção da Ferida Cirúrgica, Tratamento de Ferimentos com Pressão Negativa, Cicatrização, Cuidados de Enfermagem, Pediatria

## Abstract

**Objetivo:**

Descrever sobre o uso do tratamento de ferimentos com pressão negativa e hidrofibra com prata em um paciente pediátrico com infecção da ferida cirúrgica de difícil cicatrização.

**Método:**

Trata-se de um relato de experiência profissional de natureza descritiva sobre o uso de coberturas convencionais e do tratamento de ferimentos com pressão negativa em paciente pediátrico com infecção da ferida cirúrgica. Foi realizado no ano de 2023 em um Serviço Público de Saúde, referência no atendimento de pacientes pediátricos no Estado do Paraná.

**Resultados:**

Com 12 dias de pós-operatório de peritoneostomia, a ferida operatória iniciou com deiscência. Inicialmente, ela foi tratada com hidrofibra de prata por 22 dias e após, o tratamento de ferimentos com pressão negativa foi utilizado por 15 dias, regenerando a lesão.

**Conclusão:**

O tratamento de ferimentos com pressão negativa na pediatria mostrou-se seguro, efetivo e eficaz para o tratamento de lesão complexa e corroborou para o processo de regeneração da pele, assim como a hidrofibra com prata.

## INTRODUÇÃO

O megacolon tóxico (MT) é uma complicação intestinal que ocasiona a dilatação do cólon e fatal se não diagnosticado precocemente, pois apresenta manifestações sistêmicas de toxicidade^(1)^. É desencadeado principalmente por doenças inflamatórias intestinais (DII) como retocolite ulcerativa (RU) ou doença de Crohn (DC)^(2)^, mas também por outras etiologias não tão frequentes, como a constipação intestinal crônica, infecção por *Clostridium dificcile*, dentre outras^(3,4)^. O tratamento clínico de primeira escolha é medicamentoso, porém, em situações emergenciais como perfuração intestinal, necrose, isquemia de espessura total de parede e peritonite, o tratamento cirúrgico se faz necessário^(4)^.

A infecção de sítio cirúrgico (ISC) ocupa o 3º lugar das Infecções Relacionadas a Assistência à Saúde (IRAS), acomete 14–16% dos pacientes hospitalizados e pode ser evidenciada com até 30 dias de pós-operatório^(5,6)^. As cirurgias gastrointestinais, por explorarem cavidades já colonizadas, são consideradas potencialmente contaminadas e têm um risco maior de apresentar ISC^(7)^. Se a ferida cirúrgica estiver contaminada e apresentar deiscência de sutura, se caracteriza como lesão de difícil cicatrização^(8)^, e a terapia de feridas por pressão negativa (TFPN) é indicada para auxiliar neste tratamento^(9)^.

A TFPN começou a ser utilizada no final dos anos 80 para o tratamento de feridas crônicas e infectadas. Ela gera pressão negativa por meio de uma esponja hidrofóbica de poliuretano e um plástico filme transparente estéril aplicados no leito e bordas da ferida ligados por um tubo plástico a vácuo. Essa modalidade de curativo permite reduzir o edema e drenar o excesso de exsudato, promover o desbridamento, acelerar a perfusão, vascularização e a formação do tecido de granulação^(10,11)^.

Como há poucos estudos randomizados multicêntricos referente ao uso da TFPN na pediatria, mais pesquisas no tema são necessárias para comprovar sua eficácia em comparação a outras terapias já existentes, e assim subsidiar a tomada de decisão do enfermeiro na escolha da melhor terapia para o tratamento de lesões por ISC. No Estado do Paraná, devido ao seu elevado custo e poucas evidências, o uso desta tecnologia ainda é inexistente nas instituições terciárias de saúde da Secretaria de Saúde.

Dessa forma, o objetivo deste relato de experiência é descrever sobre o uso do tratamento de ferimentos com pressão negativa e hidrofibra com prata em um paciente pediátrico com infecção da ferida cirúrgica de difícil cicatrização.

## MÉTODO

### Desenho de Estudo

A ferramenta adotada para o desenvolvimento deste relato foi o *Standards for Reporting Qualitative Research* (SRQR)^(12)^. Os relatos de experiência não são oriundos de pesquisas. Têm por objetivo descrever a experiência individual ou de um grupo de profissionais sobre determinada situação. Sugere-se o uso do SRQR para nortear a escrita do artigo de relato de experiência, visto que ainda não há *guideline* específico com essa finalidade^(13)^.

### Local

A experiência relatada ocorreu em um hospital infantil da região metropolitana de Curitiba, Paraná. Nesta instituição pública estadual, são assistidos pacientes da faixa etária entre zero dias e 17 anos incompletos de todo o Estado, via Central de Regulação de Leitos. O referido hospital atende casos de média e alta complexidade e dispõe de 112 leitos ativos distribuídos entre: unidades de terapia intensiva (UTI) neonatal (20 leitos) e pediátrica (20 leitos), 47 leitos de enfermaria clínica e 25 leitos de enfermaria cirúrgica. Contempla ainda um centro cirúrgico com três salas operantes e um ambulatório médico de especialidades pediátricas (puericultura de baixo e alto risco, cardiologia, pneumologia, nefrologia, ortopedia, dermatologia, oftalmologia, gastroenterologia, nutrologia, otorrinolaringologia, neurologia, neurocirurgia e cirurgias vascular, plástica e cirurgia pediátrica).

A UTI Pediátrica, cenário deste relato, conta com equipe multiprofissional composta por enfermeiros, técnicos de enfermagem, médicos, médicos residentes, fisioterapeutas, nutricionista, assistente social, terapeuta ocupacional, fonoaudióloga e psicóloga para a assistência integral ao paciente.

Em 2023, a UTI Pediátrica teve 545 entradas (25 altas para casa, 491 transferências interna, 19 transferências externa e 10 óbitos), com taxa de ocupação de 83%, tempo médio de permanência de 11,7 dias e taxa de infecção de 19%, sendo 11 Infecções primária de corrente sanguínea com cateter venoso central (IPCS c/ CVC), 6 Pneumonias associadas a ventilação mecânica (PAV), três infecções do trato urinário (ITU) com cateter vesical de demora e nove outras, e taxa de mortalidade de 3,17%. As insuficiências respiratórias foram os diagnósticos mais prevalentes.

### População e Critério de Seleção

A seleção do paciente para o presente relato se deu por ter apresentado diagnóstico de MT, ser submetido a procedimento cirúrgico gastrointestinal, evoluir com ISC e apresentar deiscência de ferida operatória de difícil cicatrização, que culminou na indicação da TFPN.

### Coleta de Dados

O período de obtenção de coleta dos dados ocorreu de junho de 2023 a 27 de julho de 2023. O prontuário eletrônico foi utilizado para busca de informações sobre evolução clínica do paciente, diagnósticos e cuidados de enfermagem. As enfermeiras da Comissão de Cuidados com a Pele da Instituição elaboram um documento no Word para registro da evolução do processo cicatricial. Este relatório foi oportuno, pois os cuidados estavam descritos em um único documento, proporcionando maior rapidez de acesso para avaliação e novas tomadas de conduta. Ele continha a descrição visual da lesão, sua mensuração, registro fotográfico e cobertura utilizada para o tratamento da lesão. As enfermeiras transcreviam cronologicamente estes dados e imagens para o documento. Escala e gráfico *Bates-Jensen Wound Assessment Tool* (BWAT) também foram transcritos pelas enfermeiras para o Excel. A progenitora, que permaneceu ao lado do paciente durante toda internação, foi ativa no processo de cuidado com a lesão, assim como o paciente, após extubação.

### Análise e Tratamento dos Dados

A evolução clínica e diagnósticos de enfermagem foram analisados a partir do prontuário eletrônico. A evolução do processo cicatricial e escolha do tratamento foram analisados a partir da leitura da descrição, imagem e gráfico contidos no relatório em Word e Excel. Os preditores da ferramenta (BWAT) foram utilizados para pontuar as características da lesão e os dados do gráfico permitiram às enfermeiras visualizar se a lesão estava evoluindo para o processo cicatricial ou não. O registro fotográfico também possibilitou a análise visual das características e redução das dimensões da lesão. A análise foi realizada de maneira objetiva, descrevendo a lesão e o uso do curativo convencional e do tratamento de ferimentos compressão negativa.

### Aspectos Éticos

O projeto foi aprovado pelo Comitê de Ética em Pesquisa com Seres Humanos do Complexo Hospital de Clínicas–Universidade Federal do Paraná, conforme parecer consubstanciado número 6.159.293 de 03 de julho de 2023, respeitando os preceitos éticos da Resolução n^°^ 466/2012 do Conselho Nacional de Saúde. Houve autorização pelo responsável legal do paciente para captura e divulgação das fotos por meio da assinatura do Termo de Autorização para Uso de Imagem.

## RESULTADOS

### Relato de Experiência

Paciente P.H.F.G., sete anos, com diagnóstico de MT após biópsia de cólon em 2021. Ao longo de sua trajetória hospitalar antes da admissão no local do estudo, entre 2021 e 2023, passou por 15 laparotomias exploratórias.

Foi admitido na UTI pediátrica do hospital infantil em 01/06/2023 com distúrbio hidroeletrolítico grave em decorrência das perdas pelos estomas. Encontrava-se com bolsa de Bogotá, em jejum absoluto, sem aporte de Nutrição Parenteral Total (NPT), sob altos parâmetros de ventilação mecânica invasiva, cateter nasogástrico aberto com débito bilioso em grande quantidade. Presença de acesso venoso central duplo lúmen em subclávia direita, sedoanalgesia contínua com fentanil, midazolam e precedex.

Abdome com ferida operatória aberta, peritoniostomia e bolsa de Bogotá, cavidade evidenciando conteúdo intestinal e exsudato serossanguinolento; colostomia à esquerda edemaciada e dreno de penrose em flanco inferior direito com débito seroso, edema escrotal importante e controle de diurese em sonda vesical de demora. Extremidades frias, tempo de enchimento capilar de dois segundos, hemodinamicamente estável. Escala de Braden Q equivalente a risco moderado (13), escala de Fugulin classificatória para cuidados intensivos (44). Prontamente, foi avaliado pela equipe de cirurgia pediátrica (CIPE), que decidiu por nova abordagem cirúrgica para limpeza da cavidade abdominal, confecção de nova colostomia, fechamento da peritoniostomia e inserção de novo dispositivo de acesso vascular central.

Para cobertura da ferida operatória retroabdominal, que iniciava no apêndice xifóide e terminava em sínfise púbica, optou-se por espuma de absorção de exsudato com previsão de troca a cada dois dias se saturada. Em 07/06/2023 apresentou parada cardiorrespiratória com duração de três minutos, revertida. Iniciou uso de droga vasoativa (DVA).

Após 12 dias, com sinais de ISC, apresentou deiscência de sutura que atingiu toda parte superior e inferior à cicatriz umbilical. Houve abordagem cirúrgica com revisão da cavidade abdominal, limpeza de coágulos, desbridamento das bordas da ferida operatória e não houve aproximação das bordas, que estavam descoladas, tendendo à epibolia. No leito da ferida estava visível tecido muscular, adiposo e tecido de granulação. Pele íntegra ao redor. Abdome ainda edemaciado e tenso. A ferida operatória em quadrante inferior esquerdo, abaixo da colostomia, suturada e com dois drenos de penrose. A conduta terapêutica e cuidados de enfermagem foram: limpeza e descontaminação por meio de gazes embebidas com solução de limpeza com polihexamida, Prontosan^®^. Na sequência, as feridas foram preenchidas com hidrofibra de prata e espuma de absorção de exsudato. A periodicidade de troca deste curativo ocorreu a cada dois dias, finalizada em 12/07/2023. As dimensões das lesões em 19/06/2023 eram: ferida operatória superior 10 cm × 7 cm; inferior 6 cm × 6 cm (Figura 1). Neste dia, paciente ainda instável hemodinamicamente, em uso de DVA, com infusão de NPT, sob ventilação mecânica invasiva modo pressão controlada a volume, com pressão controlada de 21, frequência inspirada de oxigênio de 90% e saturação de oxigênio de 90%.

**Figura 1:** Pós-operatório imediato da revisão abdominal em 19/06/2023.

Em 12/07/2023, opta-se pelo uso da TFPN após discussão, orientação e explicação sobre esta tecnologia inovadora para paciente, seu responsável e equipe multidisciplinar. Paciente agora extubado, com oxigenoterapia em cateter nasal, em jejum e infusão de NPT. Os materiais foram doados para um mês de tratamento com trocas estimadas a cada quatro dias. A sequência da aplicação da TFPN aconteceu conforme as etapas a seguir: a) limpeza do leito das feridas com gaze embebida com Prontosan^®^, agindo por dez minutos; b) colocação de gaze não aderente (Jelonet) para proteger o tecido lesionado e evitar o contato do tecido com a espuma; c) aplicação da espuma, de maneira a unir as lesões superior e inferior; d) oclusão com Opsite^®^ – filme transparente e impermeável; e) conexão do Soft port; f) conexão ao compressor, numa pressão de -100 mmHg. A ferida operatória com o dreno de penrose fez um abaulamento na pele, que precisou ser vedado com pasta de hidrocoloide (Figura 2, seta verde). Nesta ferida operatória ainda havia extravasamento de grande quantidade de fluído fecal líquido devido a fístula, com a pele ao redor e abaixo apresentando lesão erosiva da epiderme. Foi utilizado pó para proteção de pele e spray barreira em toda esta área de pele. Paciente não relatou dor ou qualquer desconforto durante a aplicação desta tecnologia, nem com o vácuo. Para a equipe de enfermagem, foi um desafio conseguir manter a integridade da aderência do Opsite^®^ na pele dado o conteúdo líquido da fístula. Dimensões da lesão em 12/07/2023: lesão superior 6 cm × 3 cm; lesão inferior 4 cm × 3 cm.

**Figura 2:** Leito da ferida limpo e aplicação da terapia por pressão negativa. Na seta, pasta de hidrocoloide.

A segunda troca do sistema TFPN foi realizada dia 17/07/2023, ferida com seguintes dimensões: lesão superior 5,5 cm × 2,8 cm; lesão inferior 4 cm × 2 cm. Todo quadrante abdominal inferior esquerdo estava com aumento da área erosada pela dermatite associada ao efluente entérico da fístula. Foi iniciado banho de luz por meia hora ao dia após banho, com posterior aplicação do pó de proteção de pele com spray barreira. No leito da lesão havia tecido de granulação, com bordas aderidas (Figura 3). Foi necessário aplicar pasta de hidrocoloide em toda borda lateral esquerda do Opsite^®^ para barrar infiltração de fezes provenientes da fístula.

**Figura 3:** Lesão após limpeza com Prontosan^®^, com tecido de granulação e bordas aderidas.

A terceira troca foi realizada dia 21/07/2023: lesão superior 5 cm × 2 cm e lesão inferior 4 cm × 1 cm. O leito da lesão superior permanecia com 100% de tecido de granulação, a inferior com leito hipocorado, pois houve infiltração das fezes por debaixo do Opsite^®^ e contaminação da ferida.

A última troca do sistema TFPN aconteceu em 25/07/2023. Curativo retirado com extensa área suja e contaminada por efluente fecal. Pele ao redor com aumento da área de hiperemia e erosão da epiderme, dificultando a aderência do filme transparente. Lesão superior: 4 cm × 1,8 cm, com leito hipocorado, margens regulares e aderidas. Lesão inferior: 3 cm × 1 cm, leito hipocorado com ilhas de hipergranulação, margens regulares e aderidas ao leito. Dois dias depois, o filme transparente descolou totalmente da pele devido ao excesso de umidade e descontinuidade da epiderme, antecipando o término da terapia. A dimensão final foi: 2,5 cm × 1 cm na lesão superior e 2 cm × 0,5 cm na lesão inferior, ambas apresentando o leito opaco e hipergranulação, bordas aderidas e regulares (Figura 4). O volume total de exsudato aspirado pela tecnologia ao longo do tratamento foi de 60 mL.

**Figura 4:** Término da terapia por pressão negativa.

A continuidade dos cuidados com a lesão se deu pela limpeza com Prontosan^®^, curativo primário com espuma de transferência de exsudato com prata e curativo secundário com gazes e micropore. A pele ao redor permaneceu com uso do pó de proteção de pele e spray barreira. Paciente evoluiu com escala Braden Q 17, compatível com risco leve, Escala de Fugulin compatível com cuidados moderados, com dieta via sonda nasoenteral e infusão venosa de NPT.

O paciente recebeu alta da UTI pediátrica para a enfermaria clínica em 16/08/2023 e alta hospitalar dois dias depois, totalizando 79 dias de internação hospitalar.

O gerenciamento do processo cicatricial foi realizado pela ferramenta BWAT, como apresentado na Figura 5. Esta figura contém o escore total, somado a partir da pontuação para cada preditor de avaliação de 13 características da lesão.

**Figura 5:** Ferramenta Bates-Jansen para gerenciamento da lesão.

## DISCUSSÃO

As medidas para prevenção e controle de ISC são amplamente discutidas e desenvolvidas por órgãos competentes no âmbito nacional e nas instituições, particularmente onde estas medidas ainda são pouco evidentes na prática profissional e a tomada de decisão para manejo adequado destas complicações é mandatória no processo de cuidar da enfermagem, pois estas são lesões de difícil cicatrização^(14)^.

Algumas ações devem ser valorizadas para propiciar um cuidado de enfermagem livre de complicações em cirurgias potencialmente contaminadas. As intervenções de enfermagem que contribuem para reduzir a ISC neste perfil cirúrgico podem ser classificadas em três etapas: no pré-operatório, com uso de antibioticoterapia profilática, banho de clorexidina alcoólica e higiene das mãos; no intraoperatório, com paramentação estéril, troca de luva estéril, uso de pacote para fechamento da fáscia e da pele, degermação e antissepsia, classificação cirúrgica como potencialmente contaminada, tempo cirúrgico, redose de antibiótico, aquecimento corporal e glicemia capilar; e no pós- operatório, novamente o banho com clorexidina degermante e higiene das mãos, cuidados com curativos e drenos, controle da temperatura e da glicemia, uso de gazes esterilizadas em pacotes individuais e educação do paciente com orientações no internamento e na alta hospitalar^(15)^. Neste relato, observa-se que as múltiplas abordagens cirúrgicas do paciente, a ferida operatória aberta e a saída de conteúdo fecal, contaminando a lesão, corroboraram com a alta probabilidade de ISC. Avaliado o risco, é importante o enfermeiro escolher a melhor cobertura e realizar o curativo para propiciar o ambiente favorável para cicatrização.

Desde Florence Nightingale, o cuidado com feridas se profissionalizou e vem acumulando mais de 150 anos de conhecimento científico relacionado à cicatrização. A complexidade das lesões aumentou na mesma proporção que o avanço tecnológico de coberturas e, por conseguinte, a vasta possibilidade de coberturas pode trazer dúvidas para os enfermeiros. A premissa básica é saber que para criação de ambiente favorável à cicatrização, estes profissionais devem se apropriar da fase de cicatrização, das características da lesão e da finalidade de cada cobertura. Para melhor assertividade no cuidar de feridas, o processo de tomada de decisão (avaliação, diagnóstico, intervenção e gerenciamento da lesão, com escalas padronizadas) deve se restringir a um profissional devidamente capacitado e habilitado para esta finalidade^(16,17)^. Contudo, mesmo para profissionais qualificados, a busca por evidências no tema em pacientes pediátricos é restrita, culminando em dúvidas sobre a terapia mais efetiva^(17)^.

O processo fisiológico da cicatrização se dá em quatro fases: hemostasia, inflamação, proliferação e remodelação. Apesar da importância de todas as etapas no evento cicatricial, na inflamatória há aglomeração de macrófagos e neutrófilos, que geram exsudato e protegem a ferida de microrganismos indesejáveis e mantêm o meio propício para proliferação de novas células, remodelação da pele e cicatrização^(17)^. A escolha do curativo depende da fase de cicatrização e se modificará de acordo com sua evolução. O objetivo do curativo é manter o meio úmido, aquecido, limpo e livre da invasão bacteriana. Ele deve ser de fácil remoção (atraumático) e não demandar trocas frequentes, a fim de proporcionar estabilidade aos tecidos e maior rapidez na remodelação^(18,19)^. Como o paciente foi extubado somente no início do uso da TFPN, somente a partir deste momento ele deu seu relato sobre trocas atraumáticas e indolores.

Um dos motivos mais comuns de impedimento do processo natural da cicatrização é a infecção e a presença do biofilme, cuja formação é complexa, envolve diferentes fases e depende do tipo de microrganismo envolvido. As bactérias presentes no leito da ferida começam a se proliferar, aderir e excretar substância polimérica extracelular, a qual forma o biofilme, que por sua vez facilita a proliferação destas bactérias, acelerando a sua fase de maturação e tornando-as prontas para colonizar outras áreas^(20)^. Este sistema complexo permite não somente a proteção física, como a transferência gênica, através da qual é transmitida ao gene a sensibilidade para selecionar propriedades distintas que promovem mais proteção, auxiliam a comunicação intercelular e incentivam o crescimento de espécies benéficas. Em outras palavras, características que facilitam o melhoramento genético das bactérias, tornando-as mais resistentes ao meio e aos antibióticos, resultando em feridas difíceis de cicatriza^(21)^.

Em estudo in-vivo, foi comparado o uso da hidrofibra – isolado; hidrofibra com prata – isolado; hidrofibra + solução de limpeza composto Octenisept^®^; e hidrofibra + Prontosan^®^ e sua ação no controle do biofilme. Foi comprovado que as soluções de limpeza isentam e/ou reduzem significativamente a carga microbiana. O mesmo resultado também foi observado quando comparado ao uso de hidrofibra com prata^(22)^. O Prontosan^®^, composto por água purificada, poliaminopropil biguanida (polihexamida, PHMB) 0,1%, é um peptídeo sintético antimicrobiano que age quebrando a parede de glicossacarídeo da parede celular bacteriana, destruindo as mesmas. A betaína 0,1% contida no produto é um surfactante hidrofílico e hidrofóbico com a capacidade de realizar um desbridamento da ferida^(23)^.

Além da limpeza e antissepsia no leito da ferida, em feridas operatórias infectadas, os curativos de hidrofibra com prata promovem a hidratação ideal do meio, absorvendo o excesso de exsudato, enquanto a prata age como antimicrobiano. Este curativo também tem o benefício de não demandar trocas frequentes^(21)^. O uso destes produtos contribui para reduzir a carga microbiana local, mantendo o meio propício para cicatrização, mesmo em lesão oriunda de ferida operatória potencialmente contaminada.

Ainda que algumas modalidades terapêuticas sejam especializadas para tratar ISC, por vezes, precisam ser substituídas por tecnologias superiores, já que a presença do biofilme torna o processo cicatricial mais lento. Logo, a TFPN tem sido um itinerário terapêutico inovador e coadjuvante no tratamento de feridas de difícil cicatrização. A Organização Mundial de Saúde recomenda o uso profilático da TFPN para prevenção de ISC no pós-operatório imediato de feridas cirúrgicas de primeira intenção^(24)^. Contudo, sua utilização na prática clínica é comumente observada em complicações pós-operatórias que evoluíram com ISC^(24)^.

O mecanismo de ação da TFPN, com pressões de até -200 mmHg de maneira intermitente ou contínua no leito da lesão reduz o excesso de exsudato, o edema, a inflamação e detritos celulares. Tal ação reduz a carga bacteriana e melhora o fluxo sanguíneo e linfático, permitindo a neovascularização, melhorando o aporte de oxigênio e nutrientes para as células, formando tecido de granulação e favorecendo a epitelização e consequente cicatrização^(25,26)^.

Os desfechos significativos do uso da TFPN são as condições adversas preexistentes das lesões, como a ISC, sangramento, celulite/abscesso, septicemia e fístula, as quais somaram 63,4%. Após a instalação da terapia, estas complicações reduziram drasticamente para 6,2%^(27)^. O ônus financeiro para a instituição (profissionais, instalações, reembolso de seguros, despesas do próprio paciente) antes e após a TFPN somou, respectivamente, $11.029,01 e $3.202,43 ao mês^(9,24)^. Neste relato, uma fístula apareceu previamente à instalação da terapia, o que comprova as evidências dos estudos relatados e o desafio representado pela presença da fístula para manter a cobertura a vácuo bem aderida à pele do paciente.

Conclui-se, portanto, que a TFPN é uma tecnologia inovadora e segura para uso na pediatria e contribui para otimizar a cicatrização da ferida cirúrgica infectada.

### Limitações do Estudo

As evidências apresentadas nos estudos sobre o tratamento de ISC com uso da TFPN em cirurgias abdominais pediátricas são escassas. A vasta maioria foi conduzida em adultos. Este estudo se baseou na análise de um único cenário e de uma única amostra, pois o equipamento foi uma doação para uso exclusivo deste paciente, por um mês. Portanto, este relato de experiência é importante para suscitar nos enfermeiros pesquisas do tipo caso-controle do uso da TFPN versus terapias convencionais em outras instituições pediátricas.

### Avanços Para a Enfermagem

A produção científica de elevado nível para a TFPN realizada por enfermeiros pediatras é cada vez mais necessária e subsidiará outros profissionais em suas tomadas de decisão. Estas evidências, comprovada a sua eficácia, podem auxiliar gestores a motivar a padronização e compra deste equipamento auxiliar no processo de cicatrização. Este relato de experiência revela o protagonismo e autonomia do enfermeiro no cuidado com a pele e com feridas, fazendo dele um executante da Prática Avançada de Enfermagem, pois através da busca e geração de evidências, assume condição diferenciada para tomar decisões, condutas assertivas e assistir os pacientes que dele dependem para sua recuperação.

## CONCLUSÃO

O tratamento inicial com hidrofibra com prata durou 22 dias, com redução de seis pontos na escala BWAT na lesão superior e cinco na inferior (BWAT 33 e 31 para 27 e 26 respectivamente). A TFPN foi a terapia auxiliar, utilizada por 15 dias, com redução de 5 pontos na escala BWAT na lesão superior e 3 pontos na lesão inferior BWAT (27 e 26 para 22 e 23 respectivamente). A TFPN mostrou-se segura, eficaz e efetiva, proporcionou conforto e redução do estresse ao paciente, pelas trocas mais espaçadas, melhorando, desta forma, a experiência do paciente frente ao cuidado com feridas complexas.

A ferramenta BWAT auxilia no gerenciamento do processo cicatricial. Cada preditor das características da lesão apresenta uma variação de 1-5pontos, os quais variam de 9 (ferida em processo de regeneração) a 65 (ferida degenerada) pontos quando somados. Neste relato, todas as pontuações estavam apontando para a proximidade da regeneração.
